# Occupational Stigma Perception, Emotional Exhaustion State, and Professional Commitment Response: Understanding the Mechanisms Underlying Hotel Interns’ Perceptions of Career Prospects

**DOI:** 10.3389/fpsyg.2022.798526

**Published:** 2022-02-14

**Authors:** Lei Lei Wen, Keheng Xiang, Fan Gao, Jieling Zhou

**Affiliations:** ^1^Admissions and Employment Office, Zhejiang Technical Institute of Economics, Hangzhou, China; ^2^Graduate School, Jose Rizal University, Manila, Philippines; ^3^School of Hotel and Tourism Management, The Hong Kong Polytechnic University, Kowloon, Hong Kong SAR, China; ^4^School of Marxism, Hangzhou Medical College, Hangzhou, China

**Keywords:** occupational stigma theory, social learning theory, retention willingness, emotional exhaustion, organizational behavior

## Abstract

This study uses an integrated model of resource conservation theory and social learning theory to explore the antecedents of hotel interns’ perceptions of occupational stigma and to explore the mechanisms inherent to retention willingness. This study first manipulated relevant subjects’ experimental materials through a contextual experiment and used a one-way ANOVA to test the effects of competence stereotypes and occupational stereotypes on hotel interns’ stigma perceptions, respectively, and then used partial least squares structural equation modeling (PLS-SEM) as a statistical tool and the SmartPLS 3.0 program to validate the model of hotel interns’ occupational stigma perceptions-intention. The effects of both competence stereotypes and occupational stereotypes on hotel interns’ perceptions of occupational stigma were significant. The results of the partial least squares structural equation model showed that hotel interns’ perceptions of occupational stigma significantly contributed to emotional exhaustion and that emotional exhaustion significantly influenced hotel interns’ retention willingness, hotel interns’ perceptions of occupational stigma had a significant effect on their retention willingness, while the role of emotional exhaustion as a mediating variable and occupational commitment as a moderator. The inner psychological and behavioral linkage mechanisms of hotel interns’ occupational stigma perceptions and their retention willingness under COVID-19 were explored, and the resource dynamics operating mechanism and professional commitment were also confirmed.

## Introduction

The impact of COVID-19 on the hotel hospitality industry is incalculable, and hotels suffer from the lower numbers of guests and the challenges of human resource retention. Hotel demand for human resource planning and management has increased. For the hospitality industry, interns are a positive addition to the workforce, and a focus on interns as new human capital reduces labor costs for hotels and provides an organizational framework for building talent pipelines within hotels. Existing research has focused on generation Z as the largest proportion of hospitality workers, and studies have suggested that the existing hospitality research needs to address the changing workforce (Solnet et al., 2016), Moreover, the survival of the hospitality industry is highly dependent on a stable source of labor in the face of traditionally high and constant turnover rates and a shrinking labor market, there is a need to attract the next generation of hospitality talent (Goh and Okumus, 2020). Therefore, in the long term the hotel intern segment provides the opportunity and resource pool to observe, assess, and select regular employes. However, existing studies have shown the high turnover rate of hotel interns is a long-term challenge for the global hospitality industry, and the loss of interns increases the cost of COVID-19 human resource planning and employes’ output effectiveness in the hospitality industry. Consequently, this is detrimental to the optimization and implementation of human resource management and planning. Existing studies have conducted empirical research related to influencing factors on hotel interns’ turnover, indicating aspects like negative emotions and job stress from perceived occupational stigma can lead to low job performance and high turnover intentions among interns (Hobfoll, 1989; Pinel and Paulin, 2005). Related studies have highlighted the role of emotional exhaustion in individual turnover (Hobfoll, 2001), and more studies have explored the potential of organizational resources and occupational commitment to reduce burnout and turnover from a positive organizational behavior perspective (Blau, 1985; Chang, 1999; Cuyper et al., 2012). Existing stigma studies have empirically studied and analyzed disease stigma using COVID-19 as a lens, involving both health and non-health workers (Andaç Demirtaş-Madran, 2020; Mostafa et al., 2020) but interns, who are hospitality reservists, encountering front-line work in occupational stigma research has been neglected, and as a typical representative of non-health workers, existing stigma research needs to expand knowledge on occupational stigma research based on organizational behavior (Ramaci et al., 2021) to hotel interns who are not health workers but perform service output at high frequencies and lack a protection system.

Existing research approaches have also explored factors associated with human resource attrition in hotels concerning quantitative causal mechanism exploration and diverse influence pathways, as well as qualitative exploratory studies. However, existing research has overlooked the importance of hotel interns as a scarce resource in the hospitality industry and as individual resources in terms of talent management (Goh and Okumus, 2020). Thunnissen (2016) showed the complexity of the “black box” of hospitality industry talent management because it encourages research on the multiple levels and stakeholders involved in talent management. Thus, exploring hotel interns’ willingness to stay combines micro psychology and behavior from a positive psychology perspective where the public perspective becomes the starting point and focus of this study. Based on this, this study integrates resource retention theory and social learning theory to explore the inner mechanism of resource dynamics and the contextual interaction of social learning among hotel interns. Although few studies have examined hotel interns’ willingness to stay as a resource path for hotel human resource planning and development, this study attempts to reveal the micro-operational processes of hotel interns’ resources through the perspectives of positive organizational behavior and social psychology.

Therefore, the specific objectives of this study are as follows:

1. Determine how the intrinsic influence mechanism between hotel interns’ perceived career stigma and willingness to stay allocates and optimizes resources through the integration of resource conservation theory and social learning theory.
2. Explore the boundary condition mechanisms of different occupational stigma and emotional exhaustion levels by introducing occupational commitment as a moderating variable.
3. Taking a public perspective, determine the antecedent intrinsic influences on hotel interns’ perceptions of occupational stigma.

To address the above groundbreaking research objectives, this study combines a contextual experimental approach and quantitative analysis of causal mechanisms. These are used to validate a career stigma perception intention to stay model based on a theoretical framework integrating resource conservation theory and social learning theory. The results will inform management and internship effectiveness enhancement decisions in the post-COVID-19 hospitality industry. Moreover, it will provide a micro-behavioral and psychological theoretical reference framework for hotel interns to achieve resource self-enhancement, career planning, and psychological adjustment in the workplace from the perspective of human capital and psychological capital.

## Literature Review and Theoretical Framework

### Resource Conservation Theory

Resource conservation theory originates from stress theory, which focuses on physiological, psychological, and behavioral responses, and the most representative ones are the process-oriented stress and environmental equilibrium models (McGrath, 1976). However, the process-oriented stress model has three significant limitations. It does not provide a clear definition of individual needs and resource capacity, it does not provide a mechanistic explanation of the relationship between the two aspects, and it does not provide a standardized tool for comparing individual needs and resource capacity (Xu et al., 2021a,b). Therefore, resource conservation theory can better explain and reveal individual behavior in stressful situations (Dohrenwend and Dohrenwend, 1978). Conceptually, Hobfoll (1989) defined resources as individual characteristics, conditioned energy, and other things that make individuals feel valued, such as material resources, conditioned resources, personality traits, and energy resources. Lee and Ashforth (1996) further found that resources satisfy individual needs, and also help them in self-identification and social orientation. On the other hand, Arkin (1981) found resources have two spiral effects, the loss spiral and the value-added spiral, and thus resource conservation theory extends three theoretical corollaries: the primacy of resource conservation (Arkin, 1981). The secondary importance of resource acquisition and the creation of resource surpluses by individuals (Kobasa, 1979; Kobasa and Courington, 1981). Existing research perspectives are developed based on work situations, and in terms of resource depletion from the perspective of occupational demands. Brotheridge and Lee (2002) found that employes’ occupational demands cause resource imbalance and employes require significant internal resources for emotional regulation. Additionally, environmental factors and management styles can disrupt employes’ individual resource balance, leading to emotional depletion (Cuyper et al., 2012), It has also been shown that a lack of adequate resources (personality traits, social relationships, etc.) can lead to emotional exhaustion and affect performance (Buchwald, 2010; Barnetta et al., 2012). Some scholars have also empirically investigated resource conservation theory from the perspective of individual traits and resources. Starting with an empirical exploration of resource conservation theory, recent studies confirmed the association between individual resource characteristics and job burnout (Cordes and Dougherty, 1993); while Neil and Christopher (2012) found that individual psychological values can influence one’s resource evaluations and this influences the individual’s response to stressors. Wright and Bonett (1997) highlighted the importance of psychological well-being as a positive emotional resource, demonstrating a positive relationship between psychological well-being and performance. Hobfoll (2001) highlighted that people with more primal psychological resources have a greater ability to release stress. In summary, resource preservation theory reveals the psychological motivation of individuals to preserve, acquire, and utilize resources through the dynamic process of a resource’s intrinsic mechanism, adding new paths for the resource depletion and gain perspective to solve and reveal stress and emotional depletion problems (Tang et al., 2022).

In this study, the formation process of hotel interns’ stress is analyzed from the perspective of resource flow, and the behavioral decisions of interns under limited resources, which has predictive and explanatory power and can reveal the mechanism of hotel interns’ behavior and work outcomes.

### Social Learning Theory

Social learning theory is derived from the reinforcement learning theory of the behaviorist school, which explores the influence of individual cognition, behavior, and environmental factors and their interaction on human behavior, as well as the role of learning and self-regulation in triggering human behavior (Bandura and Walters, 1977), which highlights that people’s complex intentions and behaviors are mainly acquired, including direct and indirect knowledge (Fellnhofer, 2017). Hotel interns belong to the transitional stage from educated to working people, and they hold individual resources and processes of resource preservation and operation that are more critical in hotel work scenarios. Social learning theory emphasizes the role of hotel interns in achieving socialization, while the dynamic operational process of resources requires education to provide learning sites and knowledge (Fellnhofer, 2017). Social learning theory provides a rationale for how workplace education influences hotel interns’ willingness to stay in the workplace, career commitment, and so on. The three corollaries extended by social learning theory are interactive decision, self-regulation, and self-efficacy, highlighting the factors that influence the intrinsic mechanisms of human behavior in social learning (Bandura, 1986). Therefore, in this study, the theory explains how hotel employes’ perceived career stigma and willingness to stay are influenced by occupational commitment (competence), organizational resources (learning), and emotional exhaustion (affect), which are influenced by internal and external resources.

### Research Gap and Model Integration

**FIGURE 1:**
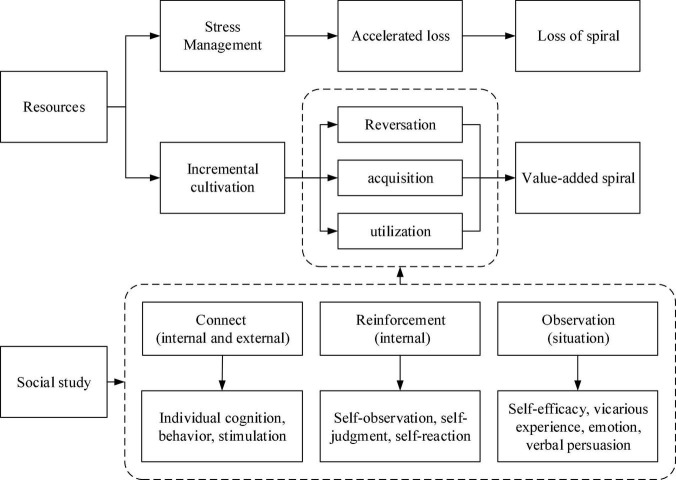
Research theoretical framework.

Resource conservation theory has been systematically and adequately argued and developed from stress theory, theoretical assumptions, core ideas, corollaries, and research perspectives. There are rich research results in positive psychology and organizational behavior. However, the existing studies mainly explain the mechanism of burnout, workplace stress, and industry performance from the perspective of individual resource input-output imbalance, but the mechanism of the inner influence of the positive side of the resource value-added spiral has not been explored sufficiently. It also provides a theoretical framework for how hotel interns achieve resource integration and optimization and high-return output through the process of social learning in this study. Therefore, this study builds the theoretical integration content framework shown in Figure 1. Combining the resource cultivation in the dynamic formation process of resource preservation theory with the process in social learning theory to realize the transformation of the efficacy of the social learning process and enhance the cultivation value of the resource value-added spiral, the theoretical framework integrated in this study therefore attempts to respond to the argumentative basis of the following research questions: First, to make up for the lack of research on the intrinsic influence mechanism and dynamic decision-making process of a resource value-added spiral in existing studies; second, the research content framework after integrating social learning theory and resource conservation theory can clarify the whole process of dynamics of hotel interns’ self-resource allocation; and third, to integrate the value orientation of the positive psychology perspective in resource conservation theory using three key nodes in social learning theory, such as association, reinforcement, and observation. This provides a reference basis for learning mechanisms in the acquisition, transformation, and integration of resources in the workplace for hotel interns in this study and provides a theoretical framework for the basis of the theoretical framework for the next research hypotheses.

### Research Hypothesis Development and Research Framework

Hughes (1962) introduced the concept related to occupational stigma, that is, dirty work, laying the foundation for the study of occupational stigma, followed by Goffman (1963) who systematically defined stigma as a label used by society to degrade and humiliate certain people or groups, divided into three main categories related to physical deficiencies associated with personality defects and signs associated with race, ethnicity, and religion. Negative occupational stigma stereotypes are widespread across industries and tend to reduce practitioners’ identity and enhance negative perceptions of their occupation (Ashforth and Kreiner, 1999; Baran et al., 2012). Existing studies on occupational stigma’s antecedents, consequences, and coping strategies have received more attention and the association between occupational stigma and the propensity to leave has been clarified. Wildes (2005) found through an empirical study in the restaurant industry that occupational stigma was associated with specific job types and that the sense of stigma associated with occupational stigma was a reason practitioners left jobs. Pinel and Paulin (2005) also concluded that employes’ awareness of occupational stigma would lead them to perceive a lack of respect at work and thus show a willingness to leave; Barbier et al. (2013) further identified professional stigma as an identity-threatening factor, where individuals perceive that the group they are embedded in will be exposed to the threat of a bad reputation, resulting in emotional exhaustion over time and a tendency to leave. From a resource conservation theory perspective, studies have confirmed the association between perceptions of occupational stigma and turnover intentions; it also found occupational stigma perceptions trigger individuals to protect resources, and some empirical studies focusing on occupational stigma outcomes highlight that employes who experience negative emotions and job stress from occupational stigma exhibit high job burnout, low job performance, and high turnover intentions (Hobfoll, 1989; Pinel and Paulin, 2005; Lai et al., 2013). Abel (2011) proposed from a psychological perspective that the constant vigilance and caution generated by occupational stigma contributes to employes’ anxiety and fear of social interactions, which further limits social interactions in the process of dressing to avoid interpersonal resource depletion, alleviate the interpersonal pressure around them, and adopt a departure coping style. It has been established that occupational stigma has negative effects on self-concept, life satisfaction and quality of professional life, stress, willingness to stay and ego (Ramaci et al., 2020). Therefore, the following hypotheses were formulated for this study on the perceived and behavioral outcomes of occupational stigma and related interns, using hotel interns as the study population. Therefore, this study takes hotel interns as the research object and proposes the following hypotheses:

***H1.*** *Hotel interns’ perceived professional stigma positively influences emotional exhaustion.*

***H2.*** *The emotional exhaustion of hotel interns negatively influences their intention to stay in their jobs.*

***H3.*** *Hotel interns’ perceived occupational stigma negatively influences their willingness to retain their jobs.*

Emotional exhaustion is a strong emotional and physiological arousal of employes in response to the great potential stress in the service industry and is also a chronic state resulting in excessive stress and depletion of employes’ psycho-emotional and behavioral well-being (Lazarus et al., 1985). Existing studies have explored the inner mechanism of the emotional exhaustion process from the perspective of the dynamic consumption of resources in resource conservation theory, for example, hotels require employes to provide quality services to customers with sincere attitudes, meaning that work itself is the process of psychological resource consumption (Brotheridge and Lee, 2002), and negative customer behaviors make employes’ work consumed resources go uncompensated, this further increases their work stress and eventually causes an imbalance of resources and generates emotional exhaustion (Koopmann et al., 2015). Therefore, employes face situations where customer stigma is imposed and employes must deplete their emotional resources to adopt effective self-motivation to maintain good performance (Harris and Reynolds, 2003). Further demonstrated that client misconduct and stigmatization can cause emotionally draining psychological responses such as anxiety and insomnia in frontline employes. Grandey et al. (2004) clarified that frontline employes are emotionally drained in relation to client abuse and that employes experience conflict when they are on duty. Therefore, when feeling personal resource depletion is not adequately compensated, there is a motivation to protect existing resources and compensate for depleted resources; emotional depletion is a good predictor of an individual’s willingness to leave (Hobfoll, 2001). Individuals who have experienced emotional depletion will defend behaviors that may produce more resource depletion and use exit strategies to protect and maintain emotional resources (Hobfoll, 2001). In summary, based on the fact that extreme customer behavior has a positive relationship with the emotional exhaustion of frontline employes in the service industry, customer stigma imposed on employes becomes a cause of emotional exhaustion (Grandey et al., 2004). And there have been reviewed studies that have explored the changes and effects of perceived occupational stigma and individual emotions with individual-level sociological theories (Andaç Demirtaş-Madran, 2020). Based on this, this study proposes the following hypothesis. Therefore, hypothesis H4 was proposed.

***H4.*** *Emotional exhaustion presents a mediating effect between perceived occupational stigma and retention willingness in hotel internships.*

Organizational factors play a role in coping with the negative impact of addressing customers, and effective organizational commitment reduces the impact of employes’ willingness to leave (Hayes, 2013). Whereas when employes encounter negative situations where their intrinsic emotions are inconsistent with the organization’s standards and they exhibit the organization’s required emotional behavior, they become burdened with the highest emotional labor levels, exacerbating individual resources. Therefore, when employes lack effective resources to control their behavior, organizational resources are an important external resource and become the main form of compensation seeking. Organizational care can maintain the balance of employes’ resources and increase their effectiveness in coping with negative situations, and organizational care can effectively reduce resource depletion caused by work stress or external non-organizational factors, such as negative violence, and alleviate emotional exhaustion, thus reducing the willingness to leave (Hobfoll, 2001). Cuyper et al. (2012) also found when individuals receive supportive resources from the organization, they become committed to and trusting of the organization, which reduces burnout and turnover. Falco et al. (2021) confirmed a stronger association between perceived risk and emotional exhaustion among employes with weak organizational commitment. Based on the reasoning of the variables in the above this study proposes the research hypothesis H5.

***H5.*** *Professional commitment moderates the relationship of perceived occupational stigma with emotional exhaustion among hotel interns.*

Professional commitment is an individual’s attitude toward a career, the intensity of motivation to engage in a selected career and is an individual commitment to a career, distinct from job embeddedness or organizational commitment (Blau, 1985). Aryee and Tan (1992) found that high professional commitment promotes employes to improve their skills and reduces willingness to leave, while Chuang et al. (2007) showed professional commitment is an important predictor of hospitality graduates’ willingness to stay as hospitality management interns. They found that when interns form a high degree of organizational socialization in hotels, they will further clarify their career plans and industry development prospects in hotels, develop a sense of identity and belong to the hospitality industry, and show a higher willingness to stay in their jobs. Ashforth et al. (1998) responded that successful organizational socialization helps employes adapt to the environment and increase their motivation and initiative: the higher the degree of socialization, the stronger the organizational identity commitment and the higher the willingness to stay. Chang (1999) verified career commitment’s role in moderating the effect of employes’ affective commitment on willingness to leave, while Luthans et al. (2007) suggested employes’ positive psychological capital is positively related to their willingness to stay and affective commitment. Falco et al. (2021), further proposed a model for the application of the job demand-resource model to workplace safety during COVID-19, which highlights the moderating effect of occupational commitment as a job resource on employes’ perceptions of emotional exhaustion and occupational expectations. Thus, hypothesis H6 was proposed.

***H6.*** *Professional commitment moderates the relationship of emotional exhaustion with hotel interns’ retention willingness.*

**FIGURE 2:**
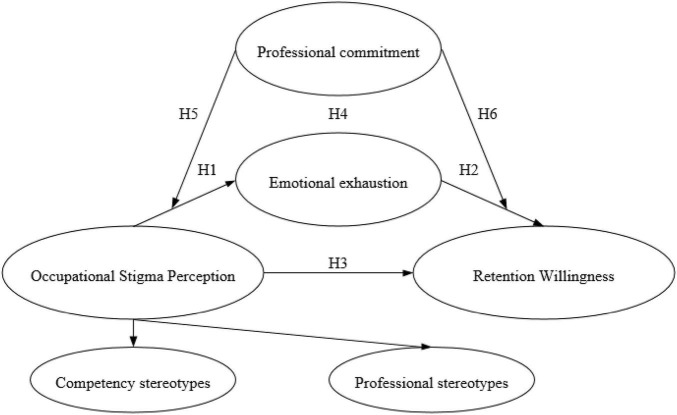
Hypothesis and content framework of this study.

Therefore, this study proposes a research hypothesis framework as shown in Figure 2.

## Research Framework

The main design of this study was divided into two situational experiments and one empirical study, both of which were conducted in a five-star hotel in Fuzhou City, Fujian Province, China. Scenario experiments I and II were designed to explore the degree of influence of ability stereotypes and professional stereotypes on perceptions of occupational stigma through a situational experiment with hotel residents; the empirical study explored the underlying mechanisms and the moderating and mediating effects of relevant variables on perceptions of occupational stigma on retention willingness through a questionnaire survey of hotel interns in this case.

Prior to the implementation of situational Experiments 1 and 2, the researchers followed research ethics by informing the participating resident respondents of the purpose of the study and the research process, and ensuring the security and privacy protection of the guests’ personal information; before the implementation of the hotel intern questionnaire, the background and content of the study were fully communicated with the interns, and the confidentiality of the questionnaire results and data during the study was ensured. All studies were approved by the research ethics committee. Based on the completion of the above preparatory work two situational experimental studies and one fieldwork study were conducted in this study in turn. Table 1 shows the research framework of this study.

**TABLE 1:** Research framework.

Study	Study Goals	Data Source	Interviewees	Experiment Manipulation
Study 1a	The effect of competency stereotypes on the perceived occupational stigma of hotel interns	Situational experiment I	Guests	Information-Competence type (Positive valence vs. Negative valence)
Study 1b	The effect of occupational stereotypes on the perceived occupational stigma of hotel interns	Situational experiment II	Guests	Information-Stereotype (Positive valence vs. Negative valence)
Study 3	Related varies of Mediating and moderating effects of perceived occupational stigma of hotel interns on their intention willingness in the workplace	Field experiment	Hotel Interns	−

### Scenario Experiments I

#### Design, Procedure, Measurement

Experiments I and II examined the effects of ability stereotypes and career stereotypes on hotel interns’ perceptions of career stigma through one-way ANOVA. This study controlled for manipulated variables related to contextual experiments to reduce intrinsic influences. The situational experiments were conducted in a real hotel setting case, and the situational cases were selected to narrate the positive and negative dimensions, respectively. The stories behind the two situational experiment cases are presented in Supplementary Appendix A. This study used convenience sampling to survey residents in a five-star hotel in Fuzhou City, Fujian Province, China. Experiments I and II sampled different batches of residents from different time periods. Respondents began filling out basic descriptive information and answering case questions online after reading the scenario cases after scanning the code to access the Golden Data platform.

The questions for the variable measures were drawn from relevant studies, and the indicators and descriptions of the specific variable measures are presented in Supplementary Appendix B. The measures of stereotypes were drawn from relevant studies (Fiske et al., 2002; Leach et al., 2007) and the measures of perceived occupational stigma were based on a relevant study by Ashforth and Kreiner (1999) based on a 7-point Likert scale (1 = strongly disagree, 7 = strongly agree) used in this study. All residents who participated in the scenario experiment were informed of the ethics of the study in advance, including privacy protection, to ensure residents were informed about the study.

### Scenario Experiment I Results

#### Pretest

Sixty valid questionnaires were collected from the pretests in this study. The pre-test randomly selected hotel guests who stayed in the hotel. The number of questionnaires in the positive and negative case groups each accounted for 50%. The results showed that 85.3% of participants believed that the cases were related to the competencies of the hotel practitioners, while 95.6% of participants believed that the cases were positive for hotel practitioners. In the negative case group, 83.2% believed the cases were related to the professional competence of hotel practitioners, and all participants believed the cases were negative for hotel practitioners; thus, Experiment I manipulated both the positive and negative dimensions of the message.

### Respondent Profile

A total of 105 valid questionnaires were returned for this study. The majority of participants in the positive case group (92.3%) believed that the descriptions of hoteliers in the cases were true, while 72.3% of the negative case group believed the cases were true; therefore, the experimental contextual material was considered acceptable.

#### Impact

The results of the independent samples *t*-test showed that the positive case group perceived significantly more than the negative case group (M_positive_ = 5.061, M_negative_ = 4.3, *t* = 2.63, *p* < 0.01) The results confirmed that positive case information significantly reduced occupational stigma perception when related to the hotelier dimension, and therefore the results support this (see Table 2).

**TABLE 2:** Test result of Experiment I.

	Valence	*n*	Stigma			Test result
			Mean	SD	MD	
Experiment I	Positive	58	5.061	0.48367	0.72762*	Support
Competence Stereotype	Negative	47	4.3333	0.7779		
Experiment I	Positive	56	5.3016	1.08163	−1.28889*	Support
Occupation Stereotype	Negative	49	6.5905	0.20805		

SD, standard deviation; MD, mean difference. *p < 0.1.

### Summary

Experiment I verified that the value of case information will significantly affect public perceived occupational stigma from the case information dimension, and that public perceived occupational stigma is less perceived in the case of positive information cases than in negative information cases. Experiment I only involved cases of hotel practitioners’ competence and did not address the effect of positive and negative dimensions of practitioners on occupational stigma perceptions; thus, Experiment II further assessed the effect of occupational descriptions on occupational stigma perceptions in terms of the positive and negative dimensions of hotel practitioners.

### Scenario Experiment II

#### Design, Procedure, Measurement

Experiment II was designed to test the effect of occupational descriptions on occupational stigma perceptions. In terms of the selection of experimental materials, the materials for Experiment II highlighted both positive and negative meanings for hotel practitioners; therefore, a pre-experiment was not necessary. As in Experiment I, Experiment II still recruited participants in the case hotel of Experiment I, using the same sampling and data collection process.

### Results of Scenario Experiment II

Similar to Experiment I, Experiment II used convenience sampling to collect data, and 105 valid data were collected. We communicated with all participants about the ethics of the study and ensured the protection of personal private information about the experiment.

The results of the independent samples *t*-test revealed that positive occupational description cases had a significant effect on reducing occupational stigma perceptions (M_positive_ = 5.6172, M_negative_ = 6.5578, *t* = 1.63, *p* < 0.01), and when occupational description information is associated with positive dimensions of hotel practitioners, positive information impressions public perceptions of occupational stigma, regardless of its value, occupational information stereotypes affect public perceptions of occupational stigma (see Table 3).

**TABLE 3:** Test result of H2.

	Valence	*n*	Stigma			Test result
			Mean	SD	MD	
Experiment II	Positive	58	4.9327	0.70613	−0.93861*	Support
Competence Stereotype	Negative	47	5.8713	0.35954		
Experiment II	Positive	56	5.6172	0.59327	−0.94059*	Support
Occupation Stereotype	Negative	49	6.5578	0.24993		

SD, standard deviation; MD, mean difference. *p < 0.01.

### Summary

Experiment II verified that from the perspective of the level of occupational descriptions. Thus, the positive or negative value of information significantly influences public occupational stigma perception. From the results of Experiment I and Experiment II, the value can significantly affect the public occupational stigma perception when case information is related to competence, as well as when case information is related to the positive dimension of occupational description, so it can be judged that information competence plays a significant role in the effect of information value on perceived occupational stigma (MD_Competence_ = −0.93861* < MD_Occuptaion_ = −0.94059*). Thus, both occupational competence and stereotypical information had a significant effect on perceived occupational stigma. Experiments I and II supported the relationship between stereotypes and perceived occupational stigma in this study, while the effect of the interaction of occupational stigma with other variables remains unclear. Therefore, this study explores the mechanism of the occurrence of mediating variables on hotel employes’ occupational stigma and intention to stay in their jobs, as well as the boundary conditions of occupational commitment as a moderating variable acting on this occurrence mechanism.

## The Partial Least Squares-Structural Equation Modeling Model Findings

### Research Analysis

The researchers examined descriptive statistics to obtain an overview of the sample by using computer program SPSS 21.0. Then, for evaluation, partial least squares structural equation modelling (PLS-SEM) was employed as the appropriate statistical tool, and the statistical approach was implemented using the Smart PLS 3.0 computer program (Duarte and Amaro, 2018). Lastly, PROCESS analysis was conducted in SPSS to examine the moderated moderation effect.

This study adopts PLS-SEM as a statistical tool and uses the Smart PLS3.0 program to implement the statistical process. PLS-SEM is a suitable method for data processing (Hayes and Preacher, 2013). Firstly, it is applicable to the moderation analysis and is better suited to dealing with constructs containing fewer items, even one or two (Hair et al., 2011, 2014). Secondly, it presents the advantages of mathematical computation and facilitates solving the small sample size requirement problem, which is suitable for mechanism analysis, theory exploration and validation (Ali et al., 2018). SPSS PROCESS is a more suitable method in the special case when researchers hypothesize based on theory that a path of both first and second stage in a mediation process is moderated.

To avoid common method bias, this study controlled for measurement items and data acquisition channels. First, it passed the Harman one-way test in SPSS 21.0, which showed that the first factor extracted explained only 33.772% of the variance, which is less than the threshold of 38.77% (Podsakoff et al., 2016). Second, following Hair et al. (2011), we examined the inner variance inflation factor (VIF) of constructs between occupational stigma perception and emotional exhaustion. The result remains 2.464, which is below 5 (Hair et al., 2011), providing information on a low level of multicollinearity. It indicates that there is unlikely to be a serious common method bias for this study.

### Measurement Scales

Occupational stigma was measured using items adapted from the study of Shantz and Booth (2014). Considering the Chinese context settings, we selected five items as loading factors for measurements. Emotional exhaustion was evaluated as Maslach (2001), which is measured by considering the specific context of a particular hotel context. The retention willingness variable was measured by following the study of Bentein et al. (2005). These items were designed to illustrate reflective indicators. The professional commitment construct was measured with the items adapted from the studies of Blau (1985) and was modified according to the hotel context. All the item statements of construct measurements used the seven-point Likert scale (1 = strongly disagree, 2 = disagree, 3 = moderately disagree, 4 = neutral, 5 = moderately agree, 6 = agree, 7 = strongly agree).

### Data Collection and Sampling

This study explored the relationship between perceived career stigma and hotel interns’ willingness to stay, and to better select the sample, this study first facilitated sampling to select hotels with five university-enterprise cooperation agreements with universities, and secondly screened case-site hotels that were implementing management trainee programs. The final case site which signed university-enterprise cooperation with five local universities and implemented three periods of management training at a five-star hotel in Fuzhou City, which has signed school-enterprise cooperation with five local universities and implemented three management trainee programs. After identifying the case sites, this study followed the following steps for data collection. First, relevant variables were identified and tested through a literature review to construct a hypothesis model. Second, based on the original scale this study checked the validity of the measurement content through multiple back-translations (Douglas and Craig, 2007). Third, the corresponding author of this study hired several research assistants to conduct the on-site survey. The research assistants explained the considerations of the questionnaire, and ensured that the hotel interns complete the questionnaire. Fourth, the data were collected from March 2021 to April 2021, 400 questionnaires were distributed through convenience sampling, invalid questionnaires were eliminated, and 348 questionnaires were finally screened (return rate of 87%). Convenience sampling (also known as availability sampling) is a specific type of non-probability sampling method that relies on data collection from population members who are conveniently available to participate in study (Saunders et al., 2012). Convenience sampling has the simplicity of sampling and convenience of research in this study, helps in research hypothesis generation, collects in a shorter period of time, and lower costs. Due to the high mobility of hotel interns and the non-uniformity of on-the-job shifts in each hotel, this study adopted convenience sampling by selecting interns on rotating shifts to fill out the questionnaire.

## Results

### Descriptive Statistics

A total of 348 (valid) hotel interns participated in this survey during the investigation period of 4 weeks (see Table 4). The proportion of male adults was approximately 40.2%. Among all the participants, the largest group constituted people at the age from 18 to 20 years, comprising 47.4% of the sample, and the second-largest group was aged between 21 and 23 years. Overall, 59.8% of the participants graduated from the major of Hospitality and Tourism. Most participants’ internship lasted for 1–3 months (34.5%), and the second largest group constituted people’s internship time lasting for 3–5 months (30.7%).

**TABLE 4:** Sample’s descriptive statistical information.

Characteristic	Valid N	Valid Percent	Cum. Percent
**Gender (valid *N* = 348)**			
Male	140	40.2	40.2
Female	208	59.8	100.0
**Age (valid *N* = 348)**			
18–20 years	166	47.4	47.7
21–23 years	76	21.8	69.5
24–25 years	48	13.8	83.3
25–26 years	58	16.7	100.0
**Internship time (valid *N* = 348)**			
0–1 months	57	16.4	16.4
1–3 months	120	34.5	50.9
3–5 months	107	30.7	81.6
5 months or above	64	18.4	100
**Graduation or study major (valid *N* = 348)**			
Hospitality and Tourism	208	59.8	59.8
Non-Hospitality and Tourism	140	40.2	100

### Analysis of Scale Reliability and Convergence Validity

Fornell and Larcker (1981) and Nunnally (1978) suggested several standards to evaluate convergence validity, such as factor loading, composite reliability, average of variance extracted (AVE), and Cronbach’s α should exceed 0.7, 0.7, 0.5, and 0.7, respectively. Moreover, R2 and path coefficients were used to explain the study model. In a PLS path model, R2 values of 0.19, 0.33, and 0.67 in a PLS path model. Chin et al. (2003) were described as weak, moderate, and substantial, respectively, by Chin (1998a, p. 323) and were associated with reflective indicators. Table 3 presents the statistical data analysis results obtained using PLS-SEM. When the factor loading of an item was <0.7, the item was deleted. This deletion led to an increase in the average variance extraction and composite reliability. The scale items S1, S2, E2, C1, and In4 were deleted because their factor loadings did not reach the specified standards.

For other scale items, the factor loadings were between 0.702 and 0.937. The R2 values of emotional exhaustion and retention willingness were 0.594, 0.177 respectively, which were the ideal predictive validity of the structural model. For “occupational stigma perception,” “emotional exhaustion,” “professional commitment,” “retention willingness,” the Cronbach’s α values were 0.739, 0.799, 0.903, and 0.817, respectively, and the composite reliability (CR) values were 0.853, 0.870, 0.923, and 0.891, respectively. The average variance extractions (AVE) were 0.627–0.732. These statistical analysis results confirm that the study model has high convergent validity. The results are presented in Table 5.

**TABLE 5:** Analysis of scale reliability and convergence validity.

Construct and scale item	Mean	SD	Loading	Cronbach’s α	CR	AVE
**Occupational stigma perception**				0.739	0.853	0.660
S1	5.97	0.998	0.588			
S2	5.96	1.036	0.650			
S3	5.65	1.096	0.702			
S4	5.67	1.182	0.774			
S5	5.73	1.206	0.844			
**Emotional exhaustion**				0.799	0.870	0.627
E1	5.72	0.958	0.715			
E2	5.85	1.074	0.608			
E3	5.55	1.008	0.776			
E4	5.67	1.190	0.753			
E5	6.02	1.339	0.869			
**Professional Commitment**				0.903	0.923	0.633
C1	5.67	1.711	0.691			
C2	6.03	1.744	0.743			
C3	5.46	1.781	0.772			
C4	5.87	1.095	0.828			
C5	5.86	1.166	0.750			
C6	5.61	1.122	0.794			
C7	5.61	1.116	0.825			
C8	6.00	1.163	0.820			
**Retention Willingness**				0.817	0.891	0.732
In1	4.76	0.702	0.913			
In2	4.51	0.783	0.937			
In3	4.3	0.784	0.931			
In4	4.57	0.867	0.596			

### Discriminant Validity

Fornell-Larcker criterion is used to determine discriminant validity (Fornell and Larcker, 1981), which compares the square root of AVE values with latent variable correlations. Satisfactory discriminant validity is evidenced by the square roots of AVE values being higher than inter-variable correlations. For the discriminant validity of the PLS model, the AVE value of each construct must be >0.5, and the AVE square root must be larger than the coefficient value of each construct pair (Fornell and Larcker, 1981). In our model (excluding negative emotions), all AVE values and AVE square roots satisfied the discriminant validity requirements. In addition to this, the study used the heterotrait-monotrait ratio of correlations (HTMT) criterion (Henseler et al., 2015) based on the multitrait-multimethod matrix, which is considered a stricter method to evaluate discriminant validity. According to Table 3, the largest HTMT value was 0.867, below the maximum cut-off of 0.9. The results are shown in Table 6.

**TABLE 6:** Discriminant validity.

	EE	RW	PC	OSP
**Fornell-Larcker Criterion**				
Emotional Exhaustion (EE)	**0.792**			
Retention Willingness (RW)	–0.243	**0.855**		
Professional Commitment (PC)	0.362	0.494	**0.796**	
Occupational Stigma perception (OSP)	0.771	0.022	0.510	**0.812**
**HTMT**				
Emotional Exhaustion (EE)				
Retention Willingness (RW)	0.312			
Professional Commitment (PC)	0.409	0.563		
Occupational Stigma perception (OSP)	0.892	0.239	0.63	

*Bold figures on the diagonal are the square roots of AVE values.*

### Path Coefficients and Mediating Analysis

Partial least squares was used to determine if the path coefficient was significant by following the re-sample procedure for *t*-testing. We adopted bootstrapping as the resampling method (Chin, 1998b). Table 7 presents the *t*-value verification results. Hypothesis H1 is true (β = 0.771, *t* = 15.452 > 1.96, and *p* < 0.001); hence, occupational stigma perception positively influences emotional exhaustion. Hypothesis H2 is true (β = −0.659, *t* = 5.962 > 1.96, and *p* < 0.001); thus, emotional exhaustion negatively influences retention. Hypothesis H3 is not true (β = 0.533, *t* = 3.315 > 1.96, and *p* < 0.01); hence, occupational stigma perception does not negatively influence retention willingness directly. The data result does now follow the previous literature that occupational stigma perception negatively influence retention willingness. We assume that instead of direct impact, occupational stigma perception negatively influence retention through emotional exhaustion.

**TABLE 7:** Path coefficients analysis.

Hypothesis	Path coefficients	*t*-Statistics
H1: Occupational Stigma Perception → Emotional Exhaustion	0.771	15.452***
H2: Emotional Exhaustion → Retention Willingness	–0.659	5.962***
H3: Occupational Stigma Perception → Retention	0.533	3.315**

**Represents p < 0.01 and ***represents p < 0.001.

**FIGURE 3:**
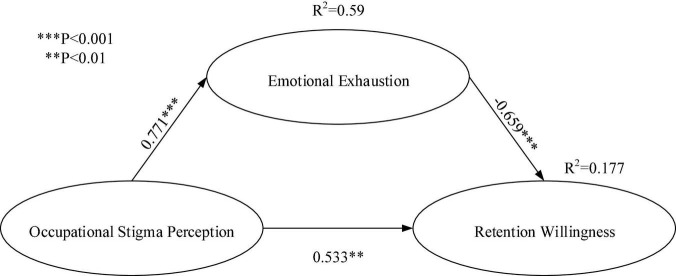
Results of the structural model.

According to the mediation analysis method proposed by Hayes (2015), a significance test was conducted via bootstrapping. Hypothesis H4 is true (the indirect effect is −0.508, and *p* < 0.001, Table 8). Occupational stigma perception was found to have significant and negative influence on retention willingness through emotional exhaustion. Figure 3 presents the results of the structural model.

**TABLE 8:** Mediating effects of OSP.

Hypothesis	Effect	*t*-Statistics
H4: Occupational Stigma perception → Emotional Exhaustion → Retention Willingness	−0.508	5.457***

***Represents p < 0.00.

### Moderated Mediation Effects

**FIGURE 4:**
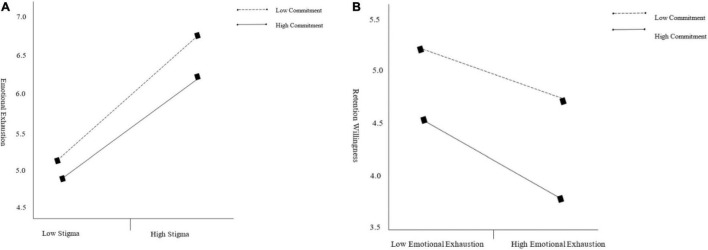
Moderating effects of professional commitment. **(A)** Moderating effects of professional commitment on the relationship between perceived occupational stigma and emotional exhaustion. **(B)** Moderating effects of professional commitment on the relationship between emotional exhaustion and retention willingness.

James and Brett (1984) coined the term “moderated mediation” to refer to a mediation relationship contingent on the moderator level. The mechanism linking X to Y is said to be conditional if the indirect effect of X on Y through M is contingent on a moderator (Hayes, 2013). Preacher et al. (2007) discussed how to analyze moderated mediation effects (i.e., conditional indirect effects), recommending bootstrapping to assess moderated mediation using a confidence interval (CI). If the 95% CI does not contain zero, then population conditional indirect effects differ significantly from zero at the 0.05 level (Preacher et al., 2007). In this study, a bootstrapping procedure with 5,000 iterations and 95% bias-corrected CIs was conducted using the SPSS PROCESS macro. The built-in Model 58 (see Hayes, 2013) was chosen as the base model for the moderated mediation model. The moderating effects of commitment were verified using hypotheses H5 and H6. Based on the path coefficient analysis, Table 9 and Figure 4 present the analysis results of the moderating effects. Table 10 presents the results of the moderated mediation effects. Bootstrapping results indicated that the conditional indirect effects of occupational stigma perception on retention willingness were significant (95% CI: [−0.4342, −0.2288]; 95% CI: [−0.4504, −0.1376], respectively). Therefore, hypothesis H5 is true (β = 0.1070, *t* = 4.3749 > 1.96, and *p* < 0.001) (95% CI [0.0589, 0.1551]). The model path diagram is shown in Figure 3.

**TABLE 9:** Moderating mediation effects of PC.

Hypothesis	Coefficients	LLCI	ULCI	*t*-Statistics
H5: Occupational Stigma Perception × Professional Commitment → Emotional Exhaustion	0.1070	0.0589	0.1551	4.3749***
H6: Emotional Exhaustion × Professional Commitment → Retention Willingness	0.0872	0.0370	0.1374	3.4171***

***Represents p < 0.001.

**TABLE 10:** Test of moderated mediation effects (conditional indirect effect).

Conditional indirect effects of Stigma on Retention willingness				
			Bootstrapping 95% CI	
Moderator level	Effect	BootSE	BootLLCI	BootULCI
Low Stigma	−0.3162	0.523	−0.4342	−0.2288
High Stigma	−0.2888	0.823	−0.4504	−0.1376

Professional commitment moderates the relationship between occupational stigma perceptions and emotional exhaustion. The high and low levels of professional commitment strengthened and weakened, respectively, and there was a positive relationship between occupational stigma perception and emotional exhaustion (Figure 4A). Hypothesis H6 is true (β = 0.0872, *t* = 3.4171 > 1.96, *p* < 0.001) (95% CI: [0.0370, 0.1374]). Professional commitment moderates the relationship between emotional exhaustion and the willingness to retain. The high and low levels of professional commitment strengthened and weakened, respectively, and there was a negative relationship between emotional exhaustion and retention willingness (Figure 4B).

## Discussion

The above study found that hotels are a stressful and emotionally risky place to work. From the beginning of internship, hotel interns are faced with a change in identity and integration into the social organization, so the entire internship process and subsequent employment is a stress management process (Lazarus and Folkman, 1984). Resource conservation theory suggests that when individuals invest many resources such as time and energy, but the return is not proportional to the investment, they suffer from burnout and other negative outcomes due to the imbalance of total resources (Hobfoll, 2001). Therefore, the empirical analysis of this study confirms that emotional exhaustion as a mediating variable significantly affects the relationship between perceived career stigma and hotel interns’ retention willingness, which reveals that behind the phenomenon of hotel interns’ emotional exhaustion, there was potential resource loss due to clients that are demanding or bullying employes. When the client performs demanding or bullying behavior toward hotel interns and projects career stigma through career stereotypes, hotel interns will perceive a strong occupational stigma, and perceived occupational stigma consumes a lot of internal resources for emotional regulation. Over time, it gradually accumulates to form emotional depletion, which ultimately affects their willingness to stay in the job due to inadequate regulation. This mediating path also demonstrated the mechanism of the influence of hotel interns’ psychological resources. This study also determined that professional commitment as a moderating variable positively moderated the relationship between perceived occupational stigma, emotional exhaustion, emotional exhaustion, and retention willingness in the hotel, respectively, which further explored the internal dynamic mechanism of the resource retention theory’s resource appreciation spiral. As an important organizational resource for hotel interns to form a higher degree of organizational socialization in hotels, they can continually self-replenish resources for themselves through organizational resources. Thereby they can further clarify their career planning development in hotels and develop a sense of identity and belonging to the hospitality industry, thus achieving greater resource increments and virtuous circles. Therefore, organizational commitment can activate the resource regeneration capacity held by hotel interns when they suffer from resource shortages and emotional depletion loads, driving hotel interns to achieve the value-added spiral effect, which diminishes perceptions of occupational stigma and enhances beliefs about job retention willingness. This study also revealed the moderating effects of high professional commitment on emotional exhaustion and perceived occupational stigma, respectively, and unexpected findings demonstrated that higher occupational commitment significantly reduced emotional exhaustion and diminishes hotel interns’ perceptions of occupational stigma. Professional commitment is different from job embeddedness or organizational commitment, as it is an individual career commitment; it is a self-storage and accumulation of resources in the medium and long term, and it is a continuous realization of psychological capital. When hotel interns consider the existing resources invested as a supplement and reserve for their future long-term career planning, they are more motivated to invest in resources, form positive work attitudes and performance behaviors, and perceive lower levels of career stigma and a more proactive approach to dismantling extreme emotions such as emotional exhaustion through available resources, which eventually leads to a lower willingness to leave and a stronger willingness to stay at the hotel. In the process of forming high professional commitment, hotel interns integrate their personal attitudes, behaviors, emotions, and environmental factors to optimize and reserve resources. These interactions ultimately result in relatively weak perceptions of career stigma, stable emotions, and willingness to stay in their jobs.

The mechanism of the effect of perceived career stigma on hotel interns’ willingness to stay in this study echoes previous research on organizational behavior, such as in the hospitality industry, in terms of the effect of perceived occupational stigma, Kreiner and Murphy (2016) confirmed that resource shortages due to stress generated by perceived career stigma increase employes’ willingness to leave, while the constant vigilance and caution generated by perceived occupational stigma motivates hotel practitioners to devote significant resources to a defensive state, further limiting normal social interactions, and they avoid interpersonal resource depletion and can only resort to leaving their jobs in response (Abel, 2011). With retention willingness level as an outcome variable, the professional commitment as a moderating variable of strong relationships proposed in this study also responds to hotel interns’ career commitment in terms of career planning and willingness to stay. In reality, hotel interns self-identify as cheap labor, lower their standards, and are less motivated to work, which eventually leads to leaving the hotel industry at the end of their internship (Nifadkar et al., 2012). Strong career commitment is also an overlapping effect of positive psychological capital, and it positively influences hotel interns’ attitude-behavior performance and reduces the negative emotion (Abbas et al., 2012). Luthans et al. (2007) also agreed that there is a positive relationship between positive psychological capital and intention to stay and affective commitment. The effect of emotional depletion as a mediating variable on perceived career stigma and intention to stay, as confirmed by this study, confirmed the resource depletion caused by the job demands perspective, the environmental factors and management style that trigger resource imbalance among interns, and the psychological stress of frontline interns caused by job insecurity and bullying by customers that disrupt individual resource balance. This highlights the significant role of emotional depletion in perceived occupational stigma and retention willingness (Cuyper et al., 2012). This study responds to Ramaci et al.’s (2021) related research on the impact of occupational stigma on organizational cognitive variables and outcomes, highlighting that occupational stigma moderates the relationship between job demands and outcomes, highlighting the importance of appropriate interventions in hotel interns, and highlighting that hotel interns are among those at high risk of being stigmatized, prompting existing research to focus on the impact of occupational stigma on frontline hotel interns’ physical and mental health effects (Wagner et al., 2014), and the perceived risk of stigmatization among hotel interns as a non-habitual environment will be valued (Xiang et al., 2021; Xu et al., 2021a), and the value added and nurturing of individual resources and the importance of career commitment in this study echoes Falco et al. (2021) for the role of work resources in the relationship between perceived risk and emotional exhaustion, and since this model of association has been little studied in past research, this study considers resources and social learning pathways as a psychological adjustment tool for hotel interns to combat emotional exhaustion and willingness to stay in their jobs, broadening the context of Di Fabio (2017) research on organizational behavioral stress in human resource management for individuals. This study expands on existing research on illness stigma by exploring the associative value of behavioral and psychological research on non-medical staff to explore the emotional and behavioral responses of hotel interns (Barattucci et al., 2020; Pagliaro et al., 2021).

### Theoretical Contributions

The theoretical contribution of this study is the conceptual framework of theoretical integration, the conditions of boundary action mechanism, and the exploration of factors influencing the antecedents of occupational stigmatization. In terms of the conceptual framework of theoretical integration, this study integrates resource conservation theory and social learning theory, which fits the applicability of hotel interns as a transition from “students” to “social beings.” Resource conservation theory is a classical theory of human organizational behavior, while social learning theory reveals human behavior and cognition in work situations through a multi-factor interaction process. Optimization, the resource-driven role of emotional depletion in the process of the mechanism of the effect of perceived career stigma on the willingness to stay in a hotel intern, reveals the social learning process driving behind professional commitment as a moderating variable, and more clearly reveals and explains the internal regulation and action mechanism of individual self-behavior and cognition and resource self-allocation caused by stress in a career stigma situation. From the perspective of resource flow and the social learning perspective of contextual interactions bring new insights into the content analysis framework of hotel interns’ resource depletion and gains, respectively. The theoretical integration model explains the process of resource self-allocation and transformation of hotel interns and highlights the mechanism of contextual interaction of social learning implied behind the highly regulative role of professional commitment. To explore boundary-action mechanism conditions, this study used career commitment as a variable in the moderating mechanism of action. Previous studies explored career commitment as an outcome variable of organizational socialization, while this study explored the association between perceived career stigma and emotional exhaustion, as well as emotional exhaustion and retention intention, by using professional commitment as a resource trait. The dynamic processes of resource flow, input, output, and transformation in professional commitment were explored, and the influence of professional commitment as a resource allocation process and value-added spiral effect in hotel interns’ occupational stigma perception and retention willingness was explained from a dynamic perspective. Professional commitment was a variable linking resource conservation theory and social learning theory provided a framework for exploring the existing segmentation of hotel interns’ positive organizational behavior. In terms of exploring the antecedent influences of occupational stigma, this study confirmed the significant effects of public stereotypes of occupational competence and occupational information on occupational stigma in hotels through contextual experiments. Previous studies explored the differential distribution patterns of occupational stigma by examining dirty jobs in hotels, confirming the significant effects of public stereotypes of occupational competence and occupational information on occupational stigma through contextual experiments and manipulated positive and negative information environments to confirm public stereotypes’ influence on hotel practitioners’ perceived occupational stigma. Moreover, it reflected the boundaries of hotel occupational stigma perceptions and the perceptual processes and effects of hotel practitioners behind the boundaries through the different public observation perspectives (Dick, 2005). This study constructs an antecedent model of information-stereotype-perception in a stigmatization context, which provides a causal mechanism basis for the subsequent cognitive behaviors of hotel interns’ acceptance of and reactions to resources after encountering a stigmatizing experience and opening the black box of hotel interns’ stigmatization perception self-mechanism.

### Practical Implications

The practical significance of this study is to provide a relevant decision basis for improving human capital effectiveness, human organization optimization, and structural framework stability in the hotel industry in the post-COVID-19 period. In terms of human capital effectiveness, when the hotel industry is experiencing a public health crisis, the emotional and psychological behaviors of hotel employes need attention, especially since the interaction between customers and hotel employes is more sensitive and vulnerable to a public health crisis, and the more sudden bullying hotel employes are subjected to it, hotels need to offer multiple case-led career awareness workshops to achieve stress management for employes, especially for hotels implementing management trainee programs, they need to reduce the loss of human capital effectiveness and enhance the risk prevention and effectiveness of human capital transformation through various forms of workshops at the early stage of interns’ induction and socialization. Regarding human organization optimization, interns are the reserve strength and useful supplement of the hotel workforce. Internship resources guarantee hotel manpower cost optimization and new strength. Therefore, the hotel needs to customize the individual development plan for interns through the design of job-level optimization and professional career planning paths, so that interns can feel the importance of the hotel’s individual career development and promote interns to establish good interpersonal relationships and be in a positive organizational culture, so that hotel interns can develop a sense of professional identity, belonging, and commitment, and then enhance their sense of self-efficacy and belonging. In terms of the human resource structural framework stability, COVID-19 underscores the fact owing to a large number of departures and rotations, hotel interns are an important organizational factor for the human resource structural framework stability. This requires hotels to pay attention to risk warning, prevention, and timeliness of talent development programs, introduce positive reward mechanisms to accelerate the organizational socialization of interns, improve their career commitment, enhance the internship experience, and ultimately form a stable working environment. The hotel needs to focus on risk warning, prevention, and the timeliness of talent development programs. Also at the level of human organizational development, hotels should encourage their organizations to optimize the balance between hotel interns’ work demands and resources (Falco et al., 2021) and considering that hotel interns need to invest additional psycho-physical energy for resource integration and social learning effectiveness, hotels should provide interns with more time and resources (e.g., equipment, adequate social space) to realize self-worth and strengthen self-efficacy (Falco et al., 2021), achieving a good balance between hotel interns’ work demands and work resources, and prevent the influence and spread of negative psychological and behavioral aspects of individuals.

### Limitations and Future Research

With the foundation of resource conservation theory and social learning theory, although this study further expands the depth of research on resources in organizational behavior and micro-psychological behavior in terms of theoretical integration, the discussion on the contextual interaction mechanism of social learning theory is insufficient, and future studies can be realized through in-depth interviews and focus group interviews with hotel interns to explore the diversified influencing factors and paths of hotel retention intentions. Second, another limitation is that the research data on occupational stigma perceptions were collected and conducted through a one-time cross-sectional data through a contextual experiment, which has certain bias interference, and the fact that most of the participants in the experiment are hotel residents also affects the results of data analysis. Third, the convenience sampling used in this study is susceptible to high levels of sampling error from selection bias and influences beyond the control of the researcher, and reliability can be questioned, making the sampling method a major limitation of this study as well. Future studies can adopt diverse research methods to improve the data quality, and improve the study’s rigor by manipulating more variables and experimental environments. Future research can focus on the combined effect of resource interaction and social learning under the professional commitment traits of hotel interns and can further theorize and analyze the specific mechanisms of resource allocation and flow at the micro-level through a deeper psychological understanding of hotel interns.

## Data Availability Statement

The original contributions presented in the study are included in the article/Supplementary Material, further inquiries can be directed to the corresponding author/s.

## Ethics Statement

The studies involving human participants were reviewed and approved by Zhejiang Technical Institute of Economics. The participants provided their written informed consent to participate in this study.

## Author Contributions

All authors made substantial contributions to conception and design, acquisition of data, or analysis and interpretation of data; took part in drafting the article or revising it critically for important intellectual content; agreed to submit to the current journal; gave final approval of the version to be published; and agreed to be accountable for all aspects of the work.

## Conflict of Interest

The authors declare that the research was conducted in the absence of any commercial or financial relationships that could be construed as a potential conflict of interest.

## Publisher’s Note

All claims expressed in this article are solely those of the authors and do not necessarily represent those of their affiliated organizations, or those of the publisher, the editors and the reviewers. Any product that may be evaluated in this article, or claim that may be made by its manufacturer, is not guaranteed or endorsed by the publisher.
