# Supplementary material for: Occupational Stigma Perception, Emotional Exhaustion State, and Professional Commitment Response: Understanding the Mechanisms Underlying Hotel Interns’ Perceptions of Career Prospects
Source: Front Psychol. 2022 Feb 14;13:798526. doi: 10.3389/fpsyg.2022.798526 (PMC8882973; doi:10.3389/fpsyg.2022.798526)
Supplement: Supplementary file 1 [file Data_Sheet_1.docx]

**Appendix A Experimental scenario**

***1. Experimental scenario 1（Information-Competence type）***

***Positive valence information***

On March 12, 2020, the front desk of a hotel encountered a check-in guest from Taiwan. After the guest presented all the documents as requested, but due to COVID-19 measures and inconsistent community and district policies and city epidemic prevention command, the guest was not able to check-in immediately. The front desk employee at that time clashed with the guest, who spoke very harsh words and denigrated the service industry. At this point, Tracy went to solve the problem as MOD. She carefully showed and compared all the policies, quickly reported to the community for advice, and then calmed the guest down and led him to a quiet area for communication. The communication process was smooth, and finally, the guest checked in without any problem.

***Negative valence information***

On February 2021, a five-star hotel had an incident where a guest checked in with another guest at the same time and only one ID card was registered. The guest was very aggressive at the time, so Sam, a front desk employee, acquiesced to the unregistered guest, and the public security department investigated the case and found that the room was not checked out under a real name. The guest took all the anger out on Sam, and Sam was also very aggrieved, and the guest was abusive. He got very worked up, and the guest had a physical confrontation.

***2. Experimental scenario 2（Information-Stereotype）***

**2.1 Positive valence information**

In March 2020, a hotel in Hangzhou, China, a hotel guest staying in the hotel suddenly had a high fever. At that point in time, the situation was not identified, and the COVID-19 policy made hotel employees very nervous.

Tim, the lobby manager, took prompt action. After analyzing the complex epidemic prevention situation, he put on protective gear and personally sent the guest to the hospital for examination. Eventually, the guests’ nucleic acid test results were negative. Tim accompanied the guest all the way through all the tests and illnesses and coordinated the PR and service teams to minimize the risk of public opinion in the hotel, which won the guest's praise, and the guest sent a thank you plaque to the hotel after he recovered.

**Negative valence information**

One such conflict occurred in a hotel in March 2021. A guest ordered takeout, but according to the epidemic prevention requirements at the time, the takeout could only be delivered to the designated location at the front desk. After the delivery, the guest wanted Peter at the front desk to send it up, but Peter explained the anti-epidemic policy and requirements to the guest and asked the guest to come downstairs and pick it up himself. The guest came downstairs in anger and was verbally abusive to Peter for three minutes and finally hit him, while Peter did not hit him back. It ended with the hotel reporting the incident.

***Appendix B Measurement***

| Dimension | Sub-dimension | Variable |
| --- | --- | --- |
| Stereotype | Competence stereotype | The staff are friendly |
|  |  | Staff are kind |
|  |  | Employees are optimistic |
|  |  | Employees are good-tempered |
|  |  | Employees are generous |
|  | Occupational stereotype | Employees are very confident. |
|  |  | Employees have good business handling skills. |
|  |  | Employees have a service philosophy of taking initiative and responsibility. |
|  |  | Employees work very efficiently. |
|  |  | Employees work well in service. |
| Stigma | Body stigma | The working environment is harsh and prone to bullying from guests. |
|  | Social stigma | Social status is not very high. |
|  | Moral stigma | Professional ethics and service consciousness need to be changed |
